# Effectiveness of brain-computer interface rehabilitation in stroke patients: A meta-analysis and restricted cubic spline analysis of randomized controlled trials

**DOI:** 10.1016/j.neurot.2026.e01086

**Published:** 2026-09-30

**Authors:** Yutian Xu, Ping Hu, Zhihui Long, Mingyu Liang, Senlin Du, Yanze Wu, Xingen Zhu, Tengfeng Yan

**Affiliations:** a Department of Neurosurgery, The Second Affiliated Hospital, Jiangxi Medical College, Nanchang University, Nanchang, Jiangxi Province, 330000, PR China; b Jiangxi Province Key Laboratory of Neurological Diseases, Nanchang, PR China; c Institute of Neuroscience, Nanchang University, Nanchang, Jiangxi Province, 330000, PR China; d Jiangxi Clinical Medical Research Center of Nervous System Diseases, Jiangxi Province, 330000, PR China

**Keywords:** Brain-computer interfaces, Stroke rehabilitation, Restricted cubic spline, Meta-analysis

## Abstract

Despite growing evidence supporting the benefits of brain-computer interface (BCI) rehabilitation in post-stroke motor recovery, this review aimed to evaluate its efficacy and determine optimal training intensity. Systematic searches were conducted across Medline, EMBASE and Web of Science from January 1, 1990, to January 30, 2026, to identify relevant RCTs. A random- or fixed-effects model was used to pool outcome data. The restricted cubic spline (RCS) method was applied to estimate the dose-response relationship between BCI training intensity and upper extremity motor function. Risk of bias was assessed using the Cochrane Risk of Bias 2 tool. Twenty-six studies, encompassing 1100 stroke patients, were included in this meta-analysis. The number of studies and participants varied across outcomes. Compared with control interventions, BCI-based rehabilitation demonstrated significant improvement in FMA-UE (24 studies, 1042 participants; MD = 1.11, 95% CI = 0.63 to 1.60), ARAT (8 studies, 524 participants; MD = 0.21, 95% CI = 0.04 to 0.39), WMFT (5 studies, 425 participants; MD = 0.94, 95% CI = 0.32 to 1.55), MBI (4 studies, 153 participants; MD = 1.17, 95% CI = 0.29 to 2.06), and BBS (2 studies, 35 participants; MD = 0.54, 95% CI = 0.02 to 1.07), but not in FMA-LE (2 studies, 78 participants; MD = −0.12, 95% CI = −0.79 to 0.55) or TUGT (2 studies, 35 participants; MD = −0.40, 95% CI = −1.15 to 0.34). Dose-response analysis suggested a non-linear, inverted U-shaped relationship between BCI rehabilitation intensity and FMA-UE, with the peak effect estimated at approximately 48 min per session. BCI-based rehabilitation may be an effective therapeutic strategy for enhancing upper-extremity motor recovery in post-stroke patients, and this review further suggests a potential optimal session duration of approximately 48 min based on dose-response analysis. However, its efficacy for lower-extremity motor recovery remains inconclusive and warrants further investigation.

## Graphical abstract

Meta-analysis results suggested that BCI-based rehabilitation serves as an effective therapeutic strategy in enhancing upper extremity motor recovery for post-stroke patients. Therapeutic efficacy follows a non-linear dose–response relationship, which means that appropriate, individualized training intensity should be adopted to optimize clinical outcomes.

**Figure:**
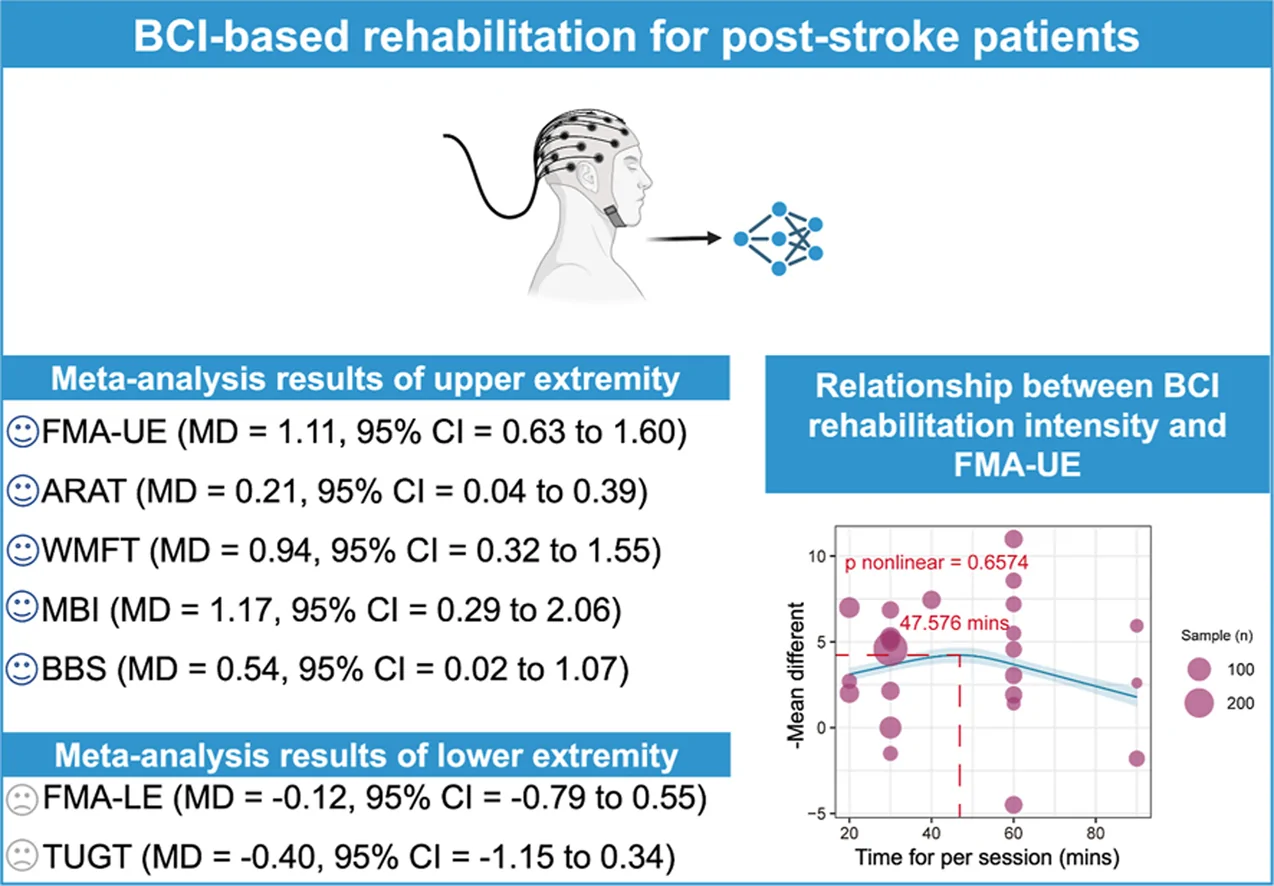

## Introduction

Stroke is a leading cause of long-term disability worldwide. Limb paralysis with hemiparesis been among its most common and devastating consequences. Approximately 50% of stroke patients experience limited dexterity, ranging from mild impairment to complete paralysis [1,2]. Despite advances in rehabilitation, up to 80% of these individuals do not achieve full functional recovery [3]. This persistent motor impairment profoundly impacts daily activities, quality of life, and the ability to return to work [4].

Intensive rehabilitation in the early weeks to months after stroke is crucial for regaining motor function [5], as this period represents a window of heightened neuroplasticity during which the brain is most capable of reorganization [6]. Conventional approaches include techniques such as mirror therapy, mental practice, and functional electrical stimulation. However, many of these interventions are resource-intensive and often accessible only in specialized clinical settings [7,8].

Although conventional rehabilitation remains essential, its effectiveness-particularly for patients with severe motor deficits-is frequently constrained by practical and physiological limitations. Consequently, there is a pressing need to develop novel therapeutic strategies that can more directly restore functional neural connectivity between motor intention and paralyzed limbs. Recently, brain-computer interface (BCI) technology represents one such promising direction for promoting motor recovery in stroke patients. BCI systems function by measuring activity from the central nervous system (CNS) and translate it into control signals for external devices, thereby aiming to replacing, restoring, enhancing, supplementing, or improve natural CNS output [9]. Early applications in rehabilitation have shown encouraging results, with studies indicating improvements in motor function for patients with stroke [10,11]. In stroke rehabilitation, BCI systems are commonly coupled with different feedback modalities. Motor-assisted feedback includes BCI-triggered functional electrical stimulation, robotic devices, exoskeletons, orthoses, or haptic devices that provide movement assistance or proprioceptive input. Visual feedback includes cursor-, screen-, virtual reality-, or graphical feedback that reinforces motor imagery or motor intention without direct physical actuation [11]. Multimodal integrated feedback combines two or more feedback channels, such as visual feedback with functional electrical stimulation, robotic assistance, or sensory stimulation. Because these feedback modalities may engage different sensorimotor mechanisms and require different clinical resources, we categorized the included studies accordingly and examined their potential influence on treatment effects [12,13].

However, while several previous investigations have compared the efficacy of BCI-based rehabilitation [14], conclusive evidence supporting its superior effectiveness for motor recovery in stroke patients, particularly for the lower extremity, remains limited. Therefore, this systematic review and meta-analysis was conducted with the primary objective of evaluating the efficacy of BCI-based rehabilitation in improving post-stroke motor function. The secondary objectives were to determine the optimal training intensity through dose-response analysis, and compare the effects of BCI rehabilitation on upper versus lower extremity motor outcomes. This synthesis seeks to clarify the current evidence base, identify potential differential effects across motor domains, and inform future clinical and research directions in neurorehabilitation.

## Method

This study was conducted according to Preferred Reporting Items for Systematic Reviews and Meta-Analyses (PRISMA) guidelines [15]. The protocol of this study has been registered in PROSPERO registry (registration no CRD420261296870).

## Search Strategy

Relevant articles were obtained from Medline, EMBASE and Web of Science database with the search algorithm: (“Brain-Computer Interfaces” OR “BCI” OR “Brain Machine Interface” OR “Motor Imagery” OR “Neurofeedback” OR “EEG-based” OR “Electroencephalography”) AND (“Stroke” OR “Cerebrovascular Accident” OR “Poststroke” OR “Post-stroke” OR “Brain Infarction” OR “Hemiplegia”). We searched for articles published between January 1, 1990, and January 30, 2026. Furthermore, published reviews and meta-analyses were reviewed, the reference lists of included studies and relevant reviews were manually screened, and forward citation tracking of key articles was conducted to complement the database search.

## Inclusion and Exclusion Criteria

Inclusion and exclusion for this study were formulated based on the PICOS principle. The detailed PICOS-based eligibility criteria were finalized before study screening began and were applied consistently throughout the screening process.

The inclusion criteria were:

(P) Patients were diagnosed with stroke without limitation of age, gender, race, geographic area, stroke type, or stroke stage.

(I) The intervention consisted of standalone BCI therapy or BCI combined with other rehabilitative modalities.

(C) Comparison treatment included no intervention or non-BCI interventions.

(O) Preestablished outcomes were: Fugl-Meyer Assessment-Upper extremity (FMA-UE), Fugl-Meyer Assessment-Lower extremity (FMA-LE), Action Research Arm Test (ARAT), Berg Balance Scale (BBS), Timed Up and Go Test (TUGT), Wolf Motor Function Test (WMFT), and Modified Barthel Index (MBI).

(S) This study merely included randomized controlled trial (RCTs).

The exclusion criteria were.

(1) Non-empirical or non-original research publications, including reviews, meta-analyses, conference abstracts, case reports, animal studies, and duplicate publications.
(2) Articles whose full text is unavailable or from which valid data cannot be extracted.
(3) Studies published in languages other than English, to ensure accuracy in data extraction and analysis and to mitigate potential errors arising from language barriers.

Title and abstract were independently reviewed by two reviewers (Xu, Long). Any disagreements were settled down with a third reviewer (Yan).

## Data Extraction and outcomes definitions

Two reviewers (Xu, Long) independently performed data extraction from included articles. Any disagreements were settled down with a third reviewer (Yan). From the eligible articles, we collected the following information: name of the first author, publication of year, country, stroke phases, interventions and control details, BCI training intensity (time for each session), rehabilitation purpose, outcomes, and follow-up.

The primary outcome was FMA-UE. The secondary outcomes were ARAT, WMFT, MBI, BBS and TUGT. Several outcomes listed in the registered protocol, including clinical response rate, neurophysiological changes, muscle strength, safety and tolerability, adherence, and acceptability, were planned for extraction. However, these outcomes were not synthesized when they were not reported, reported by only a small number of studies, or measured using heterogeneous definitions or instruments across trials. The detailed information of these outcomes definitions was presented in Supplemental Table 1.

## Quality Assessment

Since the included studies were RCTs, the methodological quality of included studies was independently evaluated by two reviewers (Xu, Long) using Cochrane Risk of Bias tool, version 2 (RoB 2.0) [16]. Any disagreements were settled down with a third reviewer (Yan). There are six dimensions: (1) Randomization process; (2) Deviations from intended interventions; (3) Missing outcome data; (4) Measurement of the outcome; (5) Selection of the reported result; (6) Overall. A final overall risk-of-bias judgment was then synthesized for each study. No restrictions were placed on the methodological quality scores during study selection. All studies that met the predefined inclusion and exclusion criteria were included for data synthesis, irrespective of their assessed risk of bias. Additionally, the overall certainty of evidence for each key outcome was assessed using the Grading of Recommendations, Assessment, Development, and Evaluation (GRADE) framework [17]. Evidence from randomized controlled trials was initially rated as high certainty and was downgraded according to five domains: risk of bias, inconsistency, indirectness, imprecision, and publication bias. Risk of bias was judged based on the RoB 2.0 assessment; inconsistency was evaluated according to the magnitude of heterogeneity and consistency of effect estimates; indirectness was assessed based on the applicability of the population, intervention, comparator, and outcomes; imprecision was evaluated according to sample size and width of the confidence intervals; and publication bias was considered based on funnel plot asymmetry, Egger’s test when applicable, and the number of available studies. The final certainty of evidence was rated as high, moderate, low, or extremely low.

## Statistical Analysis

To estimate effect size, mean difference (MD) with its 95% confidence interval (CI) was calculated for each study. Median and range data were converted to mean and standard deviations (SD) using the method described by Hozo et al. [18]. For multi-arm RCTs, we selected the comparison that most closely isolated the effect of BCI-based rehabilitation and avoided duplicate use of the same participants. Specifically, for Ang et al. (2014), the BCI-HK plus conventional rehabilitation group was compared with the HK plus conventional rehabilitation group. For Ming et al. (2025), the KI-BCI plus tDCS plus conventional rehabilitation group was compared with the tDCS plus conventional rehabilitation group.

Statistical heterogeneity among the included studies was assessed using the Cochran’s Q test (χ^2^ test) and the magnitude of heterogeneity was further quantified with the I^2^ statistic. The random-effects meta-analysis (Restricted Maximum Likelihood model) was utilized to accommodate notable heterogeneity among studies (I^2^ > 50%), preventing undue influence from any single study on our pooled estimate, or fixed-effect meta-analysis (inverse-variance model) was used (I^2^ < 50%). The Egger’s test and funnel plot were conducted to assess publication bias [19,20]. In addition, funnel plots for the primary outcome were generated separately according to external feedback type (motor-assisted feedback, visual feedback, and multimodal integrated feedback). For feedback categories containing fewer than 10 studies, the funnel plots were interpreted descriptively because of the limited reliability of assessing funnel plot asymmetry with a small number of studies. To assess whether the pooled mean differences were disproportionately influenced by any single study, a leave-one-out influence analysis was performed by successively excluding each included study and repeating the meta-analysis. To assess the robustness of the pooled findings, sensitivity analyses pre-specified in the registered protocol were performed for the primary outcome FMA-UE. First, studies judged as having an overall high risk of bias according to the Cochrane RoB 2 tool were excluded, and the meta-analysis was repeated using the remaining studies. Second, the pooled effect was re-estimated using a fixed-effect inverse-variance model to examine the influence of model assumptions. Third, an alternative random-effects approach using the Hartung-Knapp-Sidik-Jonkman method was applied to assess whether the statistical inference was robust to the choice of random-effects estimation method. Additionally, subgroup analyses were conducted to investigate potential sources of clinical and methodological heterogeneity. Subgroup variables were selected based on clinical relevance, prior BCI and stroke rehabilitation literature, and protocol characteristics that may plausibly influence treatment effects. Follow-up duration was categorized as ≤4 weeks, 5–12 weeks, or >12 weeks to distinguish short-term, medium-term, and longer-term rehabilitation periods. Session duration was categorized as 20, 30, 40, 60, or 90 min because the intervention protocols in the included trials clustered around these discrete session lengths. Other subgroup variables included external feedback type, stroke phase (chronic, subacute, or both), and stroke type (ischemic or ischemic/hemorrhagic). External feedback type was categorized as motor-assisted feedback, visual feedback, or multimodal integrated feedback. Motor-assisted feedback was defined as BCI-triggered FES, robotic, exoskeleton, orthotic, haptic, or hand-device feedback involving physical actuation or proprioceptive input. Visual feedback was defined as virtual, cursor-based, graphical, or screen-based feedback without direct physical actuation. Multimodal integrated feedback was defined as the combination of two or more feedback modalities.

Because session duration varied across studies and may have a non-linear association with treatment effect, a Restricted Cubic Spline (RCS) analysis was conducted to examine the relationship between training intensity, defined as time per training session, and the FMA-UE treatment effect. This analysis was not pre-specified in the registered protocol and was therefore interpreted as hypothesis-generating. Three knots were placed at the 25th, 50th, and 75th percentiles (0.25, 0.5, 0.75) of the distribution of time per training session to flexibly model the dose-response relationship. A likelihood ratio test was performed to compare the linear model with the RCS model, and the p-value for non-linearity was calculated to determine whether the association deviated significantly from a linear trend. A p value of <0.05 was regarded as statistically significant, except for a p value of <0.1 for the χ^2^ test. All meta-analyses were carried out using Stata 17.0. RCS analysis was conducted with the rms package in R software version 4.2.1 (R Foundation for Statistical Computing).

## Results

### Characteristics of included studies

The systematic search identified 8083 articles in total, and 2283 duplicate records were excluded. After reading titles and abstracts, 44 full texts were further reviewed. Finally, 26 eligible articles with 1100 stroke patients were included in this systematic review and meta-analysis (Fig. 1) [[21], [22], [23], [24], [25], [26], [27], [28], [29], [30], [31], [32], [33], [34], [35], [36], [37], [38], [39], [40], [41], [42], [43], [44], [45], [46]]. A detailed list of all excluded studies and the reasons for their exclusion is provided in Supplementary Table 2. All included studies were RCTs and presented acceptable methodological quality (Fig. 2). These studies were conducted in China (n = 13), Singapore (n = 3), Korea (n = 2), Germany (n = 2), Switzerland (n = 1), Denmark (n = 1), Mexico (n = 1), Russia (n = 1), Italy (n = 1), Japan (n = 1). Table 1, Supplementary Table 3 and Supplementary Table 4 presented characteristics of these included studies.

**Fig. 1:**
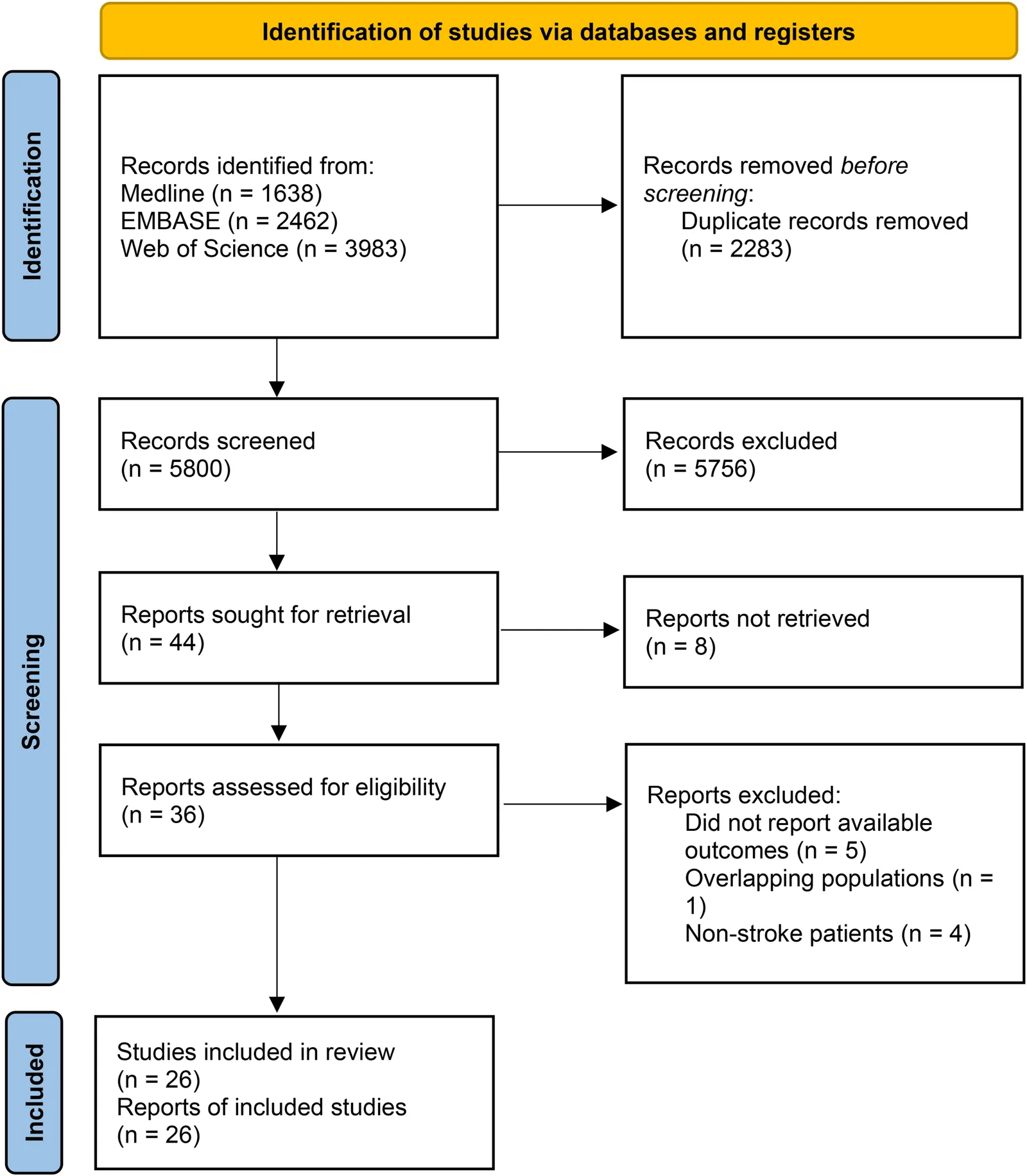
PRISMA flow diagram of the studies that were included and excluded from the systematic review and meta-analysis.

**Fig. 2:**
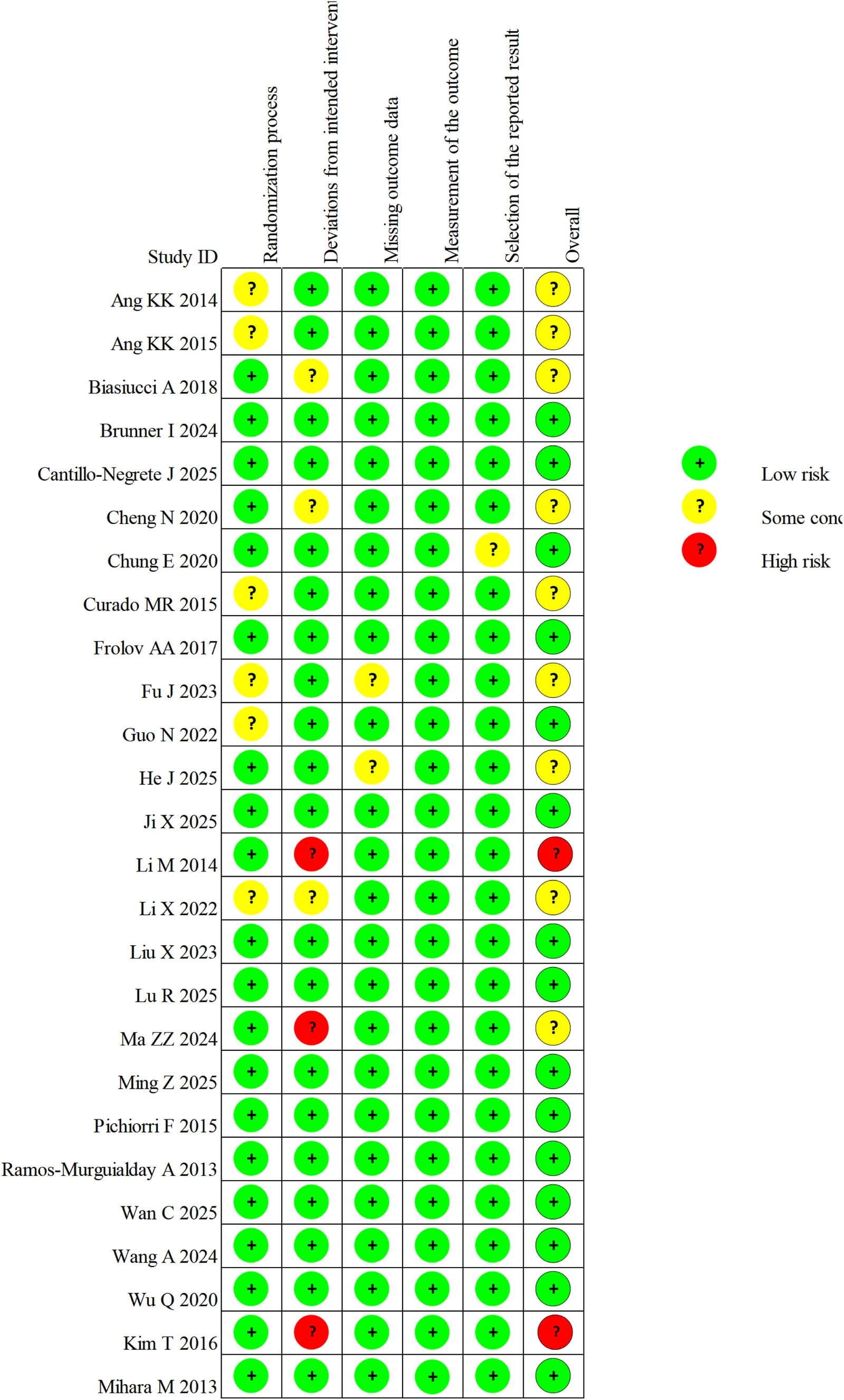
Risk of bias assessment of the included studies. Figure is available in color online only.

**Table 1:** Characteristics of the included studies.

Author & year	Country	Stroke type	Stroke phases	BCI feedback category	BCI interventions	Control interventions	Time for each session	Purpose	Outcomes	Follow-up (wks)
Ang KK 2015 [20]	Singapore	Ischemic/hemorrhagic	Chronic	Motor-assisted feedback	BCI-manus robot	Manus robot	90 min/d, 3 d/wk, 4 wks, 12 sessions	UE	dFMA-UE	28
Ang KK 2014 [19]	Singapore	Ischemic/hemorrhage	Chronic	Multimodal integrated feedback	BCI-HK + con-rehab	HK: HK + con-rehab SAT: con-rehab	60 min/d, 3 d/wk, 6 wks, 18 sessions	UE	dFMA-UE	18
Biasiucci a 2018 [21]	Switzerland	Ischemic/hemorrhagic	Chronic	Motor-assisted feedback	BCI-FES + con-rehab	Sham FES + con-rehab	60 min/d, 2 d/wk, 5wks, 10 sessions	UE	dFMA-UE	6
Brunner I 2024 [22]	Denmark	Ischemic/hemorrhage	Subacute	Multimodal integrated feedback	BCI-FES + con-rehab	Con-rehab	60 min/d, 3-4 d/wk, 3–4 wks, 12 sessions	UE	dFMA-UE, ARAT	12
Cantillo-negrete J 2025 [23]	Mexico	Ischemic/hemorrhagic	Subacute/chronic	Multimodal integrated feedback	ReHand-BCI	ReHand BCI sham mode	30 min/d, 5 d/wk, 6 wks, 30 sessions	UE	dFMA-UE	24
Cheng N 2020 [24]	Singapore	Ischemic/hemorrhage	Chronic	Motor-assisted feedback	BCI-SRG + con-rehab	SRG + con-rehab	90 min/d, 3 d/wk, 6 wks, 18 sessions	UE	dFMA-UE	24
Kim T 2016 [32]	Korea	Ischemic/hemorrhagic	Subacute/chronic	Motor-assisted feedback	AOT + BCI-FES + con-rehab	Con-rehab	30 min/d, 3 d/wk, 4 wks, 12 sessions	UE	dFMA-UE, MBI	4
Curado MR 2015 [26]	Germany	NR	Chronic	Motor-assisted feedback	BCI- robotic orthosis + con-rehab	Sham BCI + con-rehab	60 min/d, 5 d/wk, 4 wks, 20 sessions	UE	dFMA-UE	4
Frolov AA 2017 [27]	Russia	Ischemic/hemorrhagic	Subacute/chronic	Multimodal integrated feedback	BCI-exoskeleton + con-rehab	Sham BCI	30 min/d, 5 d/wk, 2 wks, 10 sessions	UE	dFMA-UE, ARAT	24
Fu J 2023 [28]	China	Ischemic/hemorrhage	Subacute/chronic	Motor-assisted feedback	BCI-hand robot + con-rehab	Con-rehab	30 min/d, 5 d/wk, 4 wks, 20 sessions	UE	dFMA-UE	4
Guo N 2022 [29]	China	Ischemic/hemorrhagic	Chronic	Motor-assisted feedback	BCI-SRG + con-rehab	Robotic: SRG control: con-rehab	60 min/d, 2 d/wks, 5wks, 10 sessions	UE	dFMA-UE, WMFT	12
He J 2025 [30]	China	Ischemic	Subacute	Multimodal integrated feedback	BCI with 8-electrode EEG enabling real-time motor intention-based feedback + con-rehab	Con-rehab	20 min/d, 5 d/wk, 2 wks, 10 sessions	UE + LE	dFMA-UE, dFMA-LE	2
Ji X 2025 [31]	China	Ischemic/hemorrhagic	Subacute	Motor-assisted feedback	BCI-SRG + con-rehab	SRG + con-rehab	60 min/d, 5 d/wk, 4wks, 20 sessions	UE	dFMA-UE, ARAT, MBI	4
Li X 2022 [33]	China	Ischemic/hemorrhagic	Subacute/chronic	Multimodal integrated feedback	BCI-exoskeleton + con-rehab	Con-rehab	60 min/d, 5 d/wk, 2 wks, 10 sessions	UE	dFMA-UE, WMFT, MBI	4
Li M 2014 [34]	China	Ischemic/hemorrhagic	Subacute	Visual feedback	BCI-FES + con-rehab	FES + con-rehab	60–90 min/d, 3 d/wk, 8 wks, 24 sessions	UE	dFMA-UE, ARAT	8
Liu X 2023 [35]	China	Ischemic/hemorrhagic	Subacute	Visual feedback	MI-BCI + con-rehab	Con-rehab	20 min/day, 5 d/wk, 3 wks, 15 sessions	UE	dFMA-UE, WMFT, MBI	3
Lu R 2025 [36]	China	NR	Chronic	Multimodal integrated feedback	Multi-FDBK BCI + con-rehab	Con-rehab	30 min/d, 5 d/wk, 4 wks, 20 sessions	UE	dFMA-UE	4
Ma ZZ 2024 [37]	China	NR	Chronic	Visual feedback	BCI + con-rehab	Con-rehab	40 min/d, 5 d/wk, 2 wks, 10 sessions	UE	dFMA-UE	2
Ming Z 2025 [38]	China	Ischemic/hemorrhagic	Subacute	Visual feedback	KI-BCI: KI BCI + con-rehab BCI-tDCS: KI-BCI + tDCS + con-rehab	tDCS: tDCS + con-rehab	30 min/d, 5 d/wk, 4 wks, 20 sessions	UE	dFMA-UE	4
Pichiorri F 2015 [39]	Italy	Ischemic/hemorrhagic	Subacute	Visual feedback	BCI-virtual feedback + con-rehab	MI + con-rehab	30 min/d, 3 d/wk, 4 weeks, 12 sessions	UE	dFMA-UE	3
Wan C 2025 [40]	China	Ischemic/hemorrhagic	Subacute/chronic	Multimodal integrated feedback	BCI + con-rehab	con-rehab	20 min/d, 5 d/wk, 4 wks, 20 sessions	LE	dFMA-LE, BBS, TUGT	4
Wang a 2024 [41]	China	Ischemic	Subacute	Multimodal integrated feedback	BCI-FES + con-rehab	con-rehab	30 min/d, 5 d/wk, 4 wks, 20 sessions	UE	dFMA-UE, ARAT, WMFT	12
Wu Q 2020 [42]	China	Ischemic/hemorrhagic	Subacute	Motor-assisted feedback	BCI-exoskeleton + con-rehab	con-rehab	60 min/d, 5 d/wk, 4 wks, 20 sessions	UE	dFMA-UE, ARAT, WMFT	4
Ramos-murguialday a 2013 [44]	Germany	NR	Chronic	Motor-assisted feedback	BCI-orthosis + con-rehab	Sham BCI + con-rehab	60 min, 5 d/wk, 4 wks, 20 sessions	UE	dFMA-UE	8
Chung E 2020 [25]	Korea	Ischemic/hemorrhagic	Chronic	Motor-assisted feedback	BCI-FES	FES	30 min/d, 3 d/wk, 5 wks, 15 sessions	LE	BBS, TUGT	5
Mihara M 2013 [43]	Japan	Ischemic/hemorrhagic	Subacute	Visual feedback	BCI-visual feedback + con-rehab	Sham BCI + con-rehab	20 min/d, 3 d/wk, 2 wks, 6 sessions	UE	dFMA-UE, ARAT	2

ARAT, Action Research Arm Test; BBS, Berg Balance Scale; BCI, brain-computer interface; CI, confidence interval; con-rehab, conventional rehabilitation; EEG, electrode electroencephalogram; FES, functional electrical stimulation; FMA-LE, Fugl-Meyer Assessment-Lower Extremity; FMA-UP, Fugl-Meyer Assessment-Upper Extremity; HK, Haptic Knob; KI, kinesthetic motor imagery; MBI, Modified Barthel Index; MI, Motor imagery; MI-BCI, motor imagery brain-computer interface; Multi-FDBK, multi-sensory feedback; SAT, standard arm therapy; SRG, Soft Robotic Glove; tDCS, transcranial direct current stimulation; TUGT, Timed Up and Go Test; WMFT, Wolf Motor Function Test; NR, not report; UE, upper extremity; LE, lower extremity.

### Primary outcome

#### Pooled results

A total of 24 studies with 1042 stroke patients reported data of FMA-UE [[21], [22], [23], [24], [25], [26], [27], [28], [29], [30], [31], [32], [33], [34], [35], [36], [37], [38], [39], [40], [41], [42], [43], [44]]. The pooled results suggested that BCI-based rehabilitation significantly improved FMA-UE compared with control treatment (MD = 1.11 (95%CI 0.63 to 1.60); I^2^ = 91.92%; Moderate quality of evidence) (Table 2, Supplementary Table 5, Fig. 3). Sensitivity analyses were conducted to assess the robustness of the primary FMA-UE finding. After excluding studies judged as having an overall high risk of bias according to RoB 2, the pooled result remained statistically significant and directionally consistent with the main analysis (MD = 1.03, 95% CI 0.54 to 1.53; I^2^ = 92.36%) (Supplementary Fig. 1). Re-analysis using a fixed-effect inverse-variance model also showed a consistent significant effect (MD = 0.67, 95% CI 0.54 to 0.79; I^2^ = 84.12%) (Supplementary Fig. 2). In addition, the Hartung-Knapp-Sidik-Jonkman random-effects approach yielded a similar conclusion (MD = 1.04, 95% CI 0.59 to 1.49; I^2^ = 91.18%) (Supplementary Fig. 3). Leave-one-out analysis demonstrated a stable feature of pooled results of FMA-UE (Supplementary Fig. 4). Nearly symmetrical funnel plot and Egger’s test (p = 0.0736 > 0.05) did not find obvious publication bias (Supplementary Fig. 5). Feedback modality-specific funnel plots for FMA-UE were generated. Egger’s tests did not detect statistically significant funnel plot asymmetry in the motor-assisted feedback (P = 0.896), visual feedback (P = 0.201), or multimodal integrated feedback (P = 0.142) subgroups. (Table 3, Supplementary Fig. 6) However, because the visual feedback and multimodal integrated feedback subgroups included fewer than 10 studies, their funnel plots and asymmetry test results should be interpreted cautiously (Supplementary Fig. 6).

**Table 2:** Pooled outcomes of included studies.

Outcomes	No. Of studies reporting variable	No. Of patients analyzed	Pooled model	Mean difference (95% CI)	I^2^ (%)	Quality of evidence^a^
Upper extremity						
FMA-UE	24	1042	Random-effects	1.11 (0.63,1.60)	91.87	Moderate
ARAT	8	524	Fixed-effect	0.21 (0.04,0.39)	0.00	High
WMFT	5	425	Random-effects	0.94 (0.32, 1.55)	81.07	Low
Activities of daily living						
MBI	4	153	Random-effects	1.17 (0.29, 2.06)	84.09	Low
Lower extremity						
FMA-LE	2	78	Random-effects	−0.12 (−0.79, 0.55)	55.28	Extremely low
BBS	2	35	Fixed-effect	0.54 (0.02,1.07)	0.00	Low
TUGT	2	35	Fixed-effect	−0.40 (−1.15, 0.34)	50.41	Low

ARAT, Action Research Arm Test; BBS, Berg Balance Scale; CI, confidence interval; FMA-LE, Fugl-Meyer Assessment-Lower Extremity; FMA-UE, Fugl-Meyer Assessment-Upper Extremity; MBI, Modified Barthel Index; TUGT, Timed Up and Go Test; WMFT, Wolf Motor Function Test.

Certainty of evidence was assessed using GRADE across risk of bias, inconsistency, indirectness, imprecision, and publication bias domains.

a , According to the GRADE framework.

**Fig. 3:**
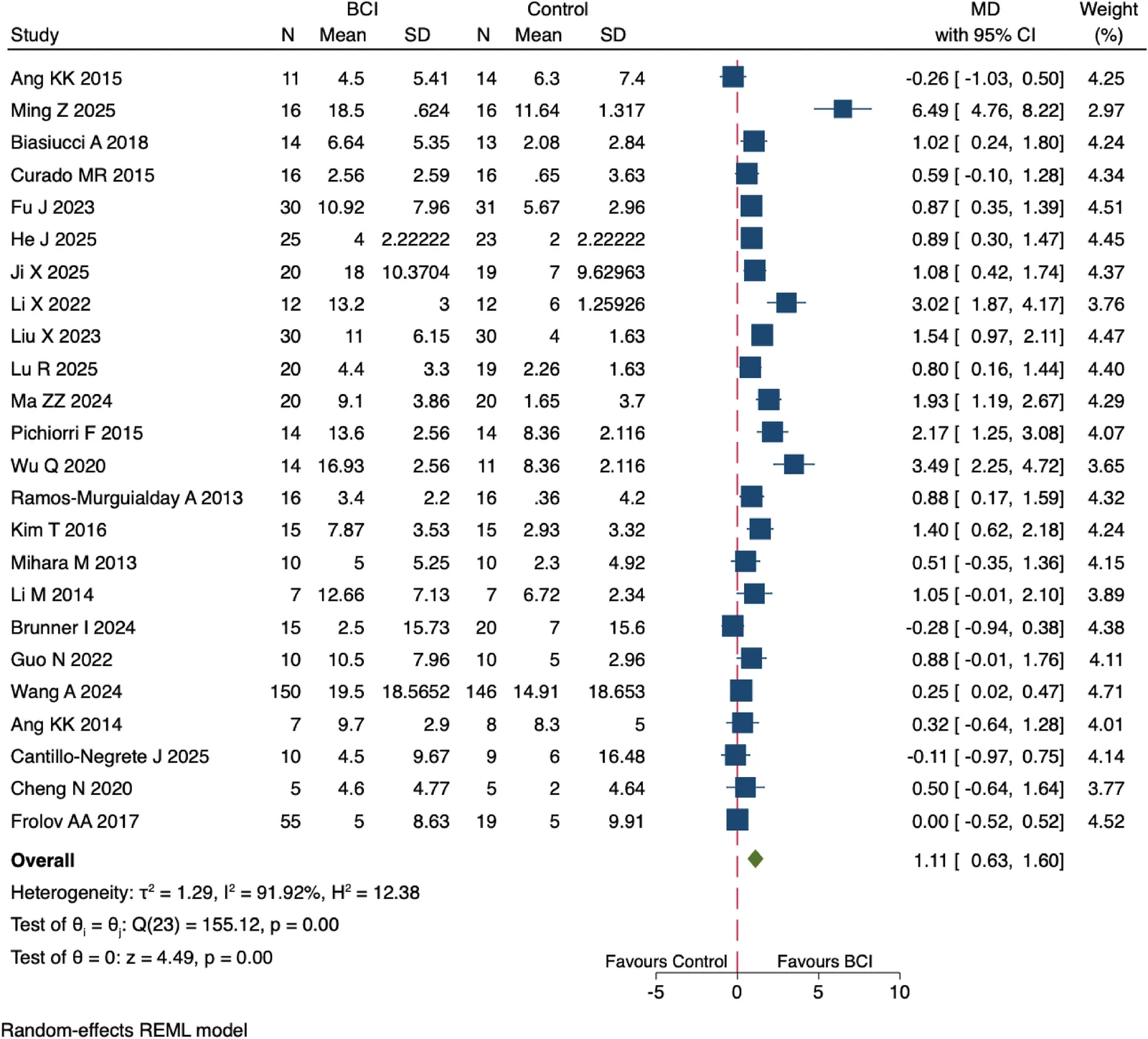
Forest plot described the pooled results of Fugl-Meyer Assessment-Upper Extremity.

**Table 3:** Subgroup analysis of Fugl-Meyer Assessment-Upper Extremity.

Outcomes	No. Of studies reporting variable	No. Of patients analyzed	Pooled model	Mean difference (95% CI)	I^2^ (%)	Quality of evidence^a^
Follow-up						
≤ 4 weeks	17	846	Random	1.42 (0.77, 2.07)	94.10	Moderate
5–12 weeks	9	451	Random	0.27 (−0.07, 0.61)	47.12	Moderate
> 12 weeks	3	44	Random	0.18 (−0.38, 0.74)	0.00	Low
Time for each session						
20 min	3	128	Random	1.03 (0.46, 1.61)	56.61	Low
30 min	8	576	Random	1.38 (0.03, 2.73)	97.69	Moderate
40 min	1	40	Random	1.93 (1.19, 2.67)	–	Low
60 min	9	249	Random	1.15 (0.41, 1.90)	86.77	Extremely low
90 min	3	49	Random	0.36 (−0.45, 1.16)	51.14	Extremely low
External feedback						
Motor-assisted feedback	10	301	Random	0.98 (0.49, 1.47)	75.13	Extremely low
Visual feedback	6	191	Random	2.18 (0.62, 3.75)	94.88	Extremely low
Multimodal integrated feedback	8	550	Random	0.54 (−0.08, 1.17)	89.02	Low
Stroke phases						
Chronic	8	230	Random	0.78 (0.35, 1.22)	61.69	Extremely low
Subacute	11	604	Random	1.51 (0.49, 2.53)	96.13	Low
Subacute/chronic	5	208	Random	0.98 (−0.07, 2.23)	90.93	Extremely low
Stroke types						
Ischemic	2	344	Random	0.51 (−0.11, 1.12)	74.92	Low
Ischemic/hemorrhagic	18	555	Random	1.23 (0.55, 1.90)	92.27	Low

CI, confidence interval.

a , According to the GRADE framework.

#### Meta-regression analysis

To assess the potential impact of study-level characteristics on treatment effects across FMA-UE outcomes and find the source of heterogeneity, we conducted a meta-regression analysis. As presented in Table 4, follow-up duration emerged as a possible source of heterogeneity. However, session duration, stroke phase, stroke type, publication year and sample size were not significantly associated with the treatment effects, indicating that these factors did not significantly alter the overall treatment effects.

**Table 4:** Meta-regression analysis for FMA-UE.

Covariate	Coefficient	Standard error	t value	p value	95% CI
Session duration	0.103	0.514	0.20	0.844	−0.981 to 1.187
Follow-up duration	−0.902	0.358	−2.52	0.022	−1.658 to −0.146
Stroke phase	0.092	0.440	0.21	0.837	−0.836 to 1.020
Stroke type	0.259	0.602	0.43	0.673	−1.012 to 1.529
Publication year	0.154	0.634	0.24	0.810	−1.183 to 1.491
Sample size	−0.641	0.648	−0.99	0.336	−2.008 to 0.726

FMA-UE, Fugl-Meyer Assessment-Upper Extremity; CI, confidence interval.

#### Subgroup analysis

Results in different subgroup were further analyzed. BCI-based rehabilitation resulted in a significant increase in FMA-UE compared with the control group at two time intervals: up to 4 weeks (MD = 1.42, 95% CI 0.77 to 2.07; I^2^ = 94.10%; Moderate quality of evidence). No significant difference between the two groups was observed at 5–12 weeks (MD = 0.27, 95% CI -0.07 to 0.61; I^2^ = 47.12%; moderate quality of evidence) or at more than 12 weeks (MD = 0.18, 95% CI -0.38 to 0.74; low quality of evidence).

Five BCI training intensities were reported in included studies. BCI-based rehabilitation led to a significant increase in FMA-UE compared with the control group when patients received 20 min rehabilitation per session (MD = 1.03, 95% CI 0.46 to 1.61; I^2^ = 56.61%; Low quality of evidence), 30 min (MD = 1.38, 95% CI 0.03 to 2.73; I^2^ = 97.69%; Moderate quality of evidence), 40 min (MD = 1.93, 95% CI 1.19 to 2.67; Low quality of evidence), 60 min (MD = 1.15, 95% CI 0.41 to 1.90; I^2^ = 86.77%; Extremely low quality of evidence). However, no significant difference was found when patients received 90 min rehabilitation (MD = 0.36, 95% CI -0.45 to 1.16; I^2^ = 51.14%; Extremely low quality of evidence) (Table 3, Supplementary Fig. 7).

Three types of BCI external feedback were recorded in included studies. Regardless of whether feedback was Motor-assisted (MD = 0.98, 95% CI 0.49 to 1.47; I^2^ = 75.13%; Extremely low quality of evidence), or visual (MD = 2.18, 95% CI 0.62 to 3.75; I^2^ = 94.80%; Extremely low quality of evidence) or multimodal integrated (MD = 0.54, 95% CI -0.08 to 1.17; I^2^ = 89.02%; Low quality of evidence), BCI-based rehabilitation conducted a significant beneficial effect on FMA-UE recovery compared with the control group (Table 3, Supplementary Fig. 8).

Three phases of stroke were reported in included studies. Regardless of whether stroke phase was chronic (MD = 0.78, 95% CI 0.35 to 1.22; I^2^ = 61.69%; Extremely low quality of evidence), or subacute (MD = 1.51, 95% CI 0.49 to 2.53; I^2^ = 96.13%; Low quality of evidence), or subacute/chronic (MD = 0.98, 95% CI -0.07 to 2.03; I^2^ = 90.93%; Extremely low quality of evidence), BCI-based rehabilitation resulted in a significant beneficial effect on FMA-UE recovery compared with the control group (Table 3, Supplementary Fig. 9).

Two types of stroke were reported across the included studies. Regardless of whether stroke type was ischemic (MD = 0.51, 95% CI -0.11 to 1.12; I^2^ = 74.92%; Low quality of evidence) or ischemic/hemorrhagic (MD = 1.23, 95% CI 0.55 to 1.90; I^2^ = 92.27%; Low quality of evidence), BCI-based rehabilitation showed a significant beneficial effect on FMA-UE recovery compared with the control group (Table 3, Supplementary Fig. 10).

#### Dose-relationship between BCI training intensity and FMA-UE

The dose-response relationship between BCI training intensity and FMA-UE was estimated using restricted cubic splines with knots located at the 0.25, 0.5, and 0.75 percentiles of the BCI training intensity. The restricted cubic spline analysis visually suggested an inverted U-shaped trend between BCI training intensity and FMA-UE, with FMA-UE peaking at 47.576 min of BCI training intensity (Fig. 4A). However, the formal test for nonlinearity was not statistically significant (p = 0.6574), indicating no evidence of a meaningful nonlinear association. Leave-one-out analysis suggested a considerable stability of restricted cubic splines (Fig. 4B).

**Fig. 4:**
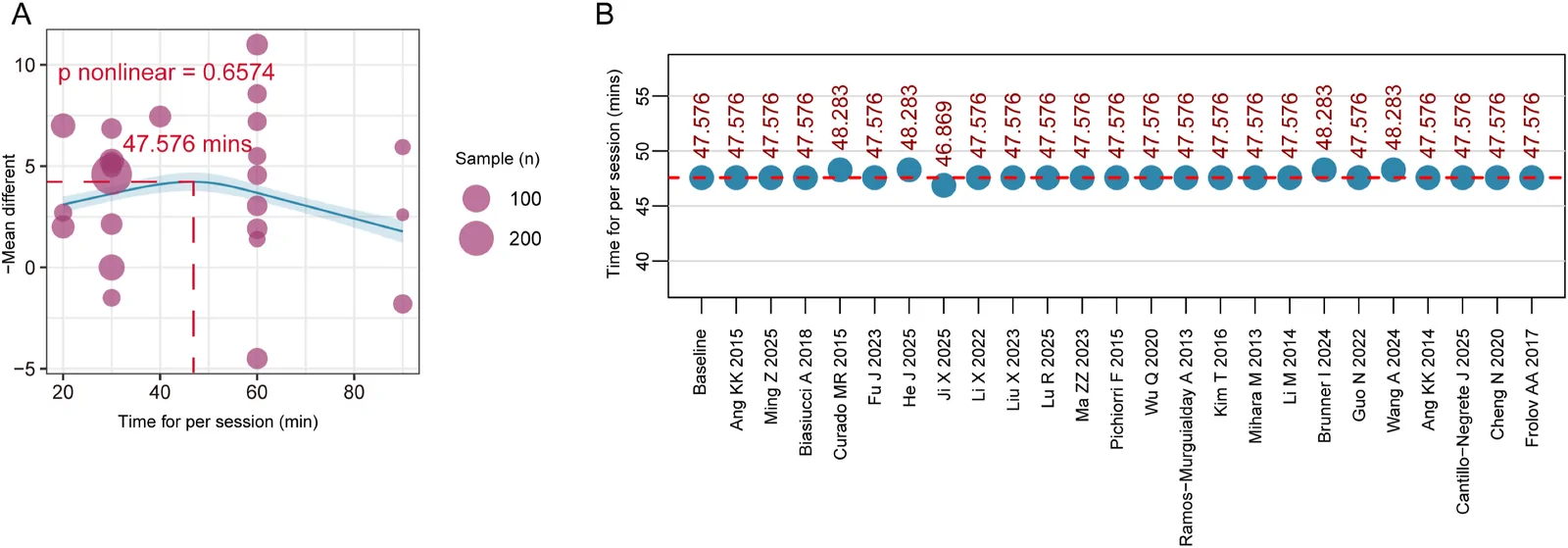
(**A**) The dose-relationship between training intensity and Fugl-Meyer Assessment-Upper Extremity was assessed with restricted cubic spline. (**B**) Leave-one-out analysis assessed the stability of restricted cubic spline.

#### Secondary outcomes

Eight studies [24,27,29,33,36,[43], [44], [45]] recorded ARAT data. BCI-based rehabilitation conducted a significant improve in ARAT compared with control group (MD = 0.21, 95% CI 0.04 to 0.39; I^2^ = 11.67%; High quality of evidence) (Table 2, Supplementary Fig. 11). Five studies [31,35,37,43,44] reported WMFT information and pooled results demonstrated a better WMFT recovery in BCI rehabilitation than control group (MD = 0.94, 95% CI 0.32 to 1.55; I^2^ = 81.07%; Low quality of evidence) (Table 2, Supplementary Fig. 12). Four studies [[33], [34], [35],^37^] reported MBI data, and the pooled results showed that BCI-based rehabilitation significantly improved ADL independence, as assessed by MBI, compared with the control group (MD = 1.17, 95% CI 0.29 to 2.06; I^2^ = 84.09%; low quality of evidence) (Table 2, Supplementary Fig. 13).

Three studies [27,32,42] compared lower extremity motor recovery. BCI-based rehabilitation significantly promoted BBS (MD = 0.54, 95% CI 0.02 to 1.07; low quality of evidence) (Table 2, Supplementary Fig. 14), but no significant effect on FMA-LE (MD = −0.12, 95% CI -0.79 to 0.55; I^2^ = 55.28%; Extremely low quality of evidence) (Table 2, Supplementary Fig. 15) and TUGT (MD = −0.40, 95% CI -1.15 to 0.34; I^2^ = 50.41%; Low quality of evidence) (Table 2, Supplementary Fig. 16).

## Discussion

This study systematically assessed the contribution of BCI-based rehabilitation interventions to motor function recovery in post-stroke patients. For the upper extremity, the pooled results indicated that BCI-based rehabilitation may be an effective strategy for enhancing motor function after stroke, with beneficial effects observed across FMA-UE, ARAT, and WMFT assessments, while MBI reflected improvement in ADL independence [[47], [48], [49]]. The RCS analysis visually suggested a possible inverted U-shaped relationship between BCI rehabilitation intensity and FMA-UE, with the largest estimated effect observed at approximately 47.576 min per session; however, this finding should be interpreted cautiously because the formal test for non-linearity was not statistically significant. As only three studies reported lower-extremity outcomes, the effectiveness of BCI-based rehabilitation for lower-extremity motor recovery remains unclear, although BBS showed a significant between-group difference [50].

The advancement of BCI embodies the critical convergence of neuroscience, artificial intelligence (AI), and bioelectronics, forging a direct link between neural activity and external technical systems [[51], [52], [53]]. Such integration allows for the efficient control of computer interfaces, assistive devices, and robotic platforms, enhancing human-machine and human-technology interaction [53,54]. These features render BCI particularly suitable for stroke rehabilitation, as it may provide a direct neural pathway to support motor relearning and functional recovery. Consequently, research in this field has expanded considerably in recent years. However, most studies to date have concentrated on upper-extremity rehabilitation, leaving the evidence base for lower-extremity applications relatively underdeveloped.

Analogous to other rehabilitation strategies, the efficacy of BCI-based rehabilitation may be influenced by several clinical and protocol-related factors, including target population, training intensity, feedback modality, and intervention duration. Our subgroup analyses showed that BCI-based rehabilitation exerted generally favorable effects on post-stroke motor recovery across different stroke types and stroke phases. The subgroup findings regarding feedback modality may also have clinical implications. Motor-assisted feedback, visual feedback, and multimodal integrated feedback were all associated with improvement in FMA-UE, suggesting that the therapeutic effect of BCI-based rehabilitation may not depend on a single feedback format. Nevertheless, these feedback modalities differ in their rehabilitation mechanisms and clinical requirements. Motor-assisted feedback, such as FES-, robotic-, exoskeleton-, orthosis-, or haptic-assisted feedback, may provide proprioceptive input and task-specific movement assistance, whereas visual feedback may mainly reinforce motor imagery or motor intention through real-time visual information [47,55,56]. Multimodal integrated feedback may theoretically provide richer sensorimotor reinforcement, although the included protocols were more heterogeneous. Moreover, some studies [10,47,56] combined BCI with other therapeutic components, such as tDCS, FES, robotic assistance, or conventional rehabilitation, which may have amplified or modified the observed treatment effects. Therefore, the pooled estimates should be interpreted as reflecting the overall effect of BCI-based rehabilitation strategies rather than the isolated effect of a single standardized BCI protocol.

Interestingly, our subgroup and RCS analyses collectively suggested that increasing training intensity was not necessarily linked to progressively better motor recovery [57,58]. This finding has direct implications for clinical practice, emphasizing the need to avoid a one-size-fits-all high-intensity strategy in stroke BCI rehabilitation. Instead, training intensity should be individualized according to stroke phase, baseline motor impairment, fatigue tolerance, cognitive ability, patient engagement, device availability, and whether BCI is combined with other interventions such as FES, robotic assistance, tDCS, or conventional rehabilitation.

While previous meta-analyses have examined the impact of BCI-based rehabilitation on post-stroke motor recovery [10,47,49], the current study remains warranted. A meta-analysis conducted by Li D et al. focused exclusively on the effectiveness of BCI rehabilitation for upper-extremity motor recovery, leaving lower-extremity outcomes unaddressed 11. By comparison, the current study included three studies [27,32,42] that reported lower-extremity data. Given the limited evidence on lower-extremity outcomes in prior research, our pooled analysis suggests that the effectiveness of BCI-based rehabilitation for lower-extremity motor recovery remains unclear, highlighting the need for more well-designed, high-quality trials. However, these findings should be interpreted with caution, as the upper and lower extremities differ substantially in rehabilitation objectives, neurophysiological mechanisms, and clinical implications. Another meta-analysis conducted by Chen H et al. included 21 studies and found that BCI training, particularly the BCI-FES paradigm, can improve upper-limb motor function in patients with stroke [59]. Therefore, our findings are largely congruent with previous reviews [11,47,59] regarding the beneficial effect of BCI-based rehabilitation on upper-extremity motor recovery [11,59], but differ by further incorporating lower-extremity outcomes [60], ADL-related outcomes, and clinically relevant moderators such as feedback modality, training intensity, and follow-up duration. The particular contribution of this review is that it not only updates the evidence supporting BCI-based rehabilitation for upper-extremity motor recovery, but also extends the synthesis to lower-extremity and ADL-related outcomes and explores clinically relevant moderators, including feedback modality, training intensity, follow-up duration, stroke phase, and stroke type. More importantly, our study reveal a potential optimal session duration of approximately 48 min based on dose-response analysis.

The confidence in the findings varied across outcomes and subgroup analyses. The evidence was relatively stronger for upper-extremity motor recovery, particularly FMA-UE, because this outcome included the largest number of studies and participants and showed a consistent beneficial direction. In contrast, lower-extremity outcomes and several subgroup analyses were based on fewer studies, smaller sample sizes, lower certainty of evidence, and residual heterogeneity, and should therefore be interpreted as preliminary [60]. Together with the revised RoB 2.0 assessment showing methodological concerns in some studies, these factors indicate that the evidence is stronger for upper-extremity motor recovery than for lower-extremity outcomes or subgroup-specific conclusions. From a clinical perspective, the pooled improvement in FMA-UE approached or exceeded commonly used Minimal Clinically Important Difference (MCID) thresholds for upper-extremity recovery after stroke, supporting the potential clinical relevance of BCI-based rehabilitation [49]. However, the clinical relevance of other outcomes should be interpreted more cautiously because MCID thresholds differ across scales, stroke stages, and patient populations, and some analyses were supported by limited evidence.

The limitations of this study should be acknowledged. Firstly, heterogeneity among the included studies cannot be overlooked. The observed heterogeneity may be explained by a number of factors, such as differences in external feedback strategies, follow-up timeframes, durations of individual training sessions, stroke phases, stroke subtypes, and combined intervention components [11]. Although we performed subgroup analyses and meta-regression to investigate potential sources of heterogeneity, substantial residual heterogeneity remained; therefore, careful interpretation of the findings is warranted [61]. High heterogeneity also lowers the certainty of evidence of our findings. Secondly, studies published in languages other than English were excluded from this study, which may lead to a degree of language bias and restrict the comprehensiveness of the evidence [50,62]. Thirdly, a number of the included studies failed to explicitly report the stroke subtypes under investigation [50]. This lack of detailed clinical baseline information may contribute to between-study heterogeneity and limit the stratification and interpretability of our findings. Fourthly, data regarding lower-extremity outcomes were reported in only three studies [27,32,42], restricting the generalizability and interpretability of the meta-analytic findings. These limitations underscore the need for future trials to report and compare more specific intervention details, including BCI paradigm, feedback modality, session duration, total training dose, combination with other interventions, and follow-up timing [63]. Future studies should also include larger samples, report baseline motor impairment in detail, evaluate lower-extremity outcomes more consistently, and incorporate clinically interpretable indices such as MCID-based responder analyses when possible [64].

## Conclusion

This meta-analysis demonstrates that BCI-based rehabilitation is an effective strategy for upper extremity motor function recovery in stroke patients. Importantly, therapeutic efficacy follows a non-linear dose-response relationship, indicating that an appropriate, individualized training intensity should be adopted to optimize outcomes. However, the current evidence does not support a definitive effect on lower extremity motor recovery. Further high-quality trials focusing on lower limb function to establish its efficacy and optimal application parameters is critical needed.

## Ethics statement

This meta-analysis is based on retrospective, observational data derived from prior studies that obtained full ethical approval. As the data are completely de-identified and anonymized, their secondary use for the present analysis does not require separate ethical approval.

## Availability of data and materials

This study is a meta-analysis that utilized data extracted from previously published studies. All data used in this analysis are available in the original publications. No new primary data were generated or collected for this study.

## Author contributions

Conception and design: Yan, Zhu. Acquisition of data: Xu,Hu, Du, Wu, Long. Analysis and interpretation:Xu, Liang. Drafting the article:Xu, Hu, Long. Critically revising the article: Yan, Zhu. Reviewed submitted version of manuscript: Yan, Zhu, Xu, Hu, Long, Liang, Wu, Du. Approved the final version of the manuscript on behalf of all authors: Yan. Statistical analysis:Xu, Hu. Administrative/technical/material support: Yan, Zhu. Study supervision: Yan, Zhu.

## Funding

This work was supported with 10.13039/501100001809National Natural Science Foundation of China (No. 82403635 for TFY). 10.13039/501100004479Natural Science Foundation of Jiangxi Province (No. 20232BAB206085 and 20224BAB216074 for TFY), 10.13039/501100010031Postdoctoral Research Foundation of China (No. 2022M721452 for TFY). Jiangxi Provincial Clinical Research Center for Neurological Diseases, China (No.2023BCG74009 for XGZ). Jiangxi Key Laboratory of Neurological Diseases (No.2024SSY06071 for XGZ).

## Competing interests

The authors report no conflict of interest concerning the materials or methods used in this study or the findings specified in this paper.

## List of abbreviations

ARAT: Action Research Arm Test
BBS: Berg Balance Scale
BCI: brain-computer interface
CI: confidence interval; con-rehab, conventional rehabilitation
EEG: electrode electroencephalogram
FES: functional electrical stimulation
FMA-LE: Fugl-Meyer Assessment-Lower Extremity
FMA-UE: Fugl-Meyer Assessment-Upper Extremity
HK: Haptic Knob
KI: kinesthetic motor imagery
MBI: Modified Barthel Index
MI: Motor imagery
MI-BCI: motor imagery brain-computer interface
Multi-FDBK: multi-sensory feedback
SAT: standard arm therapy
SRG: Soft Robotic Glove
tDCS: transcranial direct current stimulation
TUGT: Timed Up and Go Test
WMFT: Wolf Motor Function Test
NR: not report
UE: upper extremity
LE: lower extremity
RCS: restricted cubic spline.
