# Supplementary material for: Effectiveness of brain-computer interface rehabilitation in stroke patients: A meta-analysis and restricted cubic spline analysis of randomized controlled trials
Source: Neurotherapeutics. 2026 Sep 30;23(6):e01086. doi: 10.1016/j.neurot.2026.e01086 (PMC13654521; doi:10.1016/j.neurot.2026.e01086)
Supplement: Supplementary file 1 [file mmc1.docx]

Supplementary Table 1. Detailed description of outcomes.

| Outcomes | Definitions |
| --- | --- |
| Upper extremity |  |
| FMA-UE | A standardized scale (0–66) assessing motor recovery of the upper limb after stroke. Higher scores indicate better motor function. The score change (post-intervention minus baseline) reflects the improvement in upper limb motor function and recorded for this systematic review and meta-analysis. |
| ARAT | A performance test (0–57) evaluating upper limb functional ability, particularly grasp, grip, pinch, and gross movements. Higher scores denote better function. The score change quantifies the improvement in upper limb functional capacity after rehabilitation and recorded for this systematic review and meta-analysis. |
| WMFT | A timed and functional ability scale (0–75) assessing upper extremity motor function through 15 tasks. Higher scores indicate better performance. The score change represents the gain in motor performance and efficiency following intervention and recorded for this systematic review and meta-analysis. |
| MBI | A measure (0–100) of independence in activities of daily living. Higher scores reflect greater independence. The score change indicates the improvement in daily living independence from baseline to post-rehabilitation and recorded for this systematic review and meta-analysis. |
| Lower extremity |  |
| FMA-LE | A measure (0–100) of independence in activities of daily living. Higher scores reflect greater independence. The score change indicates the improvement in daily living independence from baseline to post-rehabilitation and recorded for this systematic review and meta-analysis. |
| BBS | A 14-item scale (0–56) assessing static and dynamic balance abilities. Higher scores indicate better balance. The score change reflects the improvement in balance performance from pre- to post-intervention and recorded for this systematic review and meta-analysis. |
| TUGT | A timed test (in seconds) measuring dynamic balance and mobility by assessing the time taken to stand up, walk 3 meters, turn, walk back, and sit down. Lower times indicate better mobility. The time change (reduction in seconds) indicates improved mobility and balance efficiency and recorded for this systematic review and meta-analysis. |

ARAT, Action Research Arm Test; BBS, Berg Balance Scale; FMA-LE, Fugl-Meyer Assessment-Lower Extremity; FMA-UP, Fugl-Meyer Assessment-Upper Extremity; MBI, Modified Barthel Index; TUGT, Timed Up and Go Test; WMFT, Wolf Motor Function Test.

Supplementary Table 2. Full-text articles excluded after eligibility assessment and reasons for exclusion

| First author，year | The reason of exclusion |
| --- | --- |
| Chen L, 2021^[1]^ | Did not report available outcomes |
| Mane R, 2019^[2]^ | Did not report available outcomes |
| Rodríguez-García ME, 2025^[3]^ | Did not report available outcomes |
| Jang YY, 2016^[4]^ | Did not report available outcomes |
| Ang KK, 2015^[5]^ | Did not report available outcomes |
| Ma ZZ, 2023^[6]^ | Overlapping populations |
| Kumari R, 2024^[7]^ | Non-stroke patients |
| Miao Y, 2020^[8]^ | Non-stroke patients |
| Fateeva VV, 2023^[9]^ | Non-stroke patients |
| Müller-Putz GR 2014^[10]^ | Non-stroke patients |

Supplementary Table 3. Underlying BCI paradigms and baseline motor impairment characteristics of the included studies.

| Study | BCI paradigm | Baseline Severity |
| --- | --- | --- |
| Ang KK 2014 | Motor Imagery (MI) | Moderate-to-severe (FMMA 10 – 50, baseline 27.0 ± 13.8) |
| Ang KK 2015 | MI | Moderate-to-severe (FMMA 4 – 40, baseline BCI 26.3 ± 10.3 / 26.4 ± 14.8) |
| Biasiucci A 2018 | Motor Attempt (MA) | Severe (FMA-UE ≤ 40, baseline 21.6 ± 10.8 / 19.9 ± 11.2) |
| Brunner I 2024 | MI | Severe (ARAT < 13; median ARAT 0, median FMA-UL ~4) |
| Cantillo-Negrete J 2025 | MI | Moderate-to-severe (FMA-UE 24.5 [20, 36] / 26 [16, 37.5], ARAT 8.5 [5, 26] / 3 [1.8, 30.5]) |
| Cheng N 2020 | MI | Moderate-to-severe (chronic stroke with hand impairment, FMA-UE 10–45 ) |
| Chung E 2020 | Attention/Concentration-based BCI | Moderate-to-severe (chronic hemiparetic stroke with foot drop) |
| Curado MR 2015 | MI | Severe (FMA-hand 3.29 ± 0.51, no active finger extensions) |
| Frolov AA 2017 | MI | Severe (FMMA 19.5 [11.0; 40.0], ARAT 4.5 [0.0; 30.0]) |
| Fu J 2023 | MA | Moderate-to-severe (Brunnstrom I–V) |
| Guo N 2022 | SSVEP | Moderate-to-severe (FMA-UL 5-50, baseline FMA-UL ~15: Control 14.4 ± 8.7 / Robotic 14.9 ± 5.2 / BCI-robotic 14.9 ± 8.0) |
| He J 2025 | MA/MI | Moderate-to-severe (ischemic stroke with motor impairment; proximal upper limb muscle strength 1–3; baseline FMA-UE median 23.0 [11.0–46.0] / 18.0 [11.0–29.0]) |
| Ji X 2025 | MI | Moderate-to-severe (Brunnstrom hand II–IV, left hemiparesis, baseline FMA-UL median 22.0 [18.5–27.0] / 21.0 [19.0–25.0], ARAT median 3.0 [3.0–6.0] / 5.0 [3.0–7.0]) |
| Kim T 2016 | MI + Action Observation | Moderate (first-time stroke <12 months, FMA-UE impaired, baseline 26.8 ± 7.2 / 21.87 ± 8.22) |
| Li M 2014 | MI | Severe (Brunnstrom III–V, severe upper extremity paralysis, baseline FMA 9–22, ARAT mostly 0) |
| Li X 2022 | SMR-BCI with audio-cue, motor observation & multisensory feedback | Moderate-to-severe (Brunnstrom < IV, hand paralysis, FMA-UE 22.6 ± 13.7 / 24.3 ± 17.1) |
| Liu X 2023 | MI | Moderate-to-severe (Brunnstrom UL III–V, hand II–V, FMA-UE 29.0 [21.8, 42.3] / 36.0 [24.0, 43.3]) |
| Lu R 2025 | MI with multi‑modal sensory feedback | Severe (FMA‑UE < 31; baseline FMA-UE 13.90 ± 6.97 / 13.68 ± 6.28) |
| Ma ZZ 2024 | MI | Moderate-to-severe (chronic hemiplegia with UL motor dysfunction) |
| Mihara M 2013 | MI | Moderate-to-severe (FMA 20.7 ± 13.8 / 24.0 ± 13.8, ARAT 15.0 ± 20.3 / 14.7 ± 18.2) |
| Ming Z 2025 | MI | Moderate-to-severe (FMA-UE KI-BCI 22.00 ± 0.49 / tDCS 22.29 ± 1.75 / BCI-tDCS 22.57 ± 3.26; ARAT KI-BCI 15.93 ± 2.33 / tDCS 15.07 ± 2.22 / BCI-tDCS 12.93 ± 2.65) |
| Pichiorri F 2015 | MI | Severe (FMA 23.4 ± 17.3 / 24.2 ± 18.2, hemiplegia / hemiparesis, first-ever stroke 6wk–6mo) |
| Ramos-Murguialday A 2013 | MI | Severe (cFMA 12.15 ± 8.8, no residual finger movements) |
| Wan C 2025 | MI+VR (dual-task) | Moderate-to-severe (Brunnstrom ≤ IV, lower limb, FMA-LE 17.29 ± 5.25 / 20.50 ± 6.63) |
| Wang A 2024 | MI | Moderate-to-severe (NIHSS arm item 1–3; FMA-UE 28 [18–45] / 31 [16–52], ARAT 14.00 [4.00–35.00] / 13.50 [3.00–40.50]) |
| Wu Q 2020 | MI | Moderate-to-severe (Brunnstrom ≤ IV, UL paralysis,FMA-UL 18.43 ± 2.65 / 14.09 ± 2.51; ARAT 9.50 [3.00,23.25] / 1.00 [0.00,10.00]; WMFT 30.07 ± 3.38 / 25.09 ± 2.96) |

Note: Baseline data are presented according to the following rules: if full-sample (overall) baseline statistics were reported in the original study, they are presented directly; if only grouped data were available, they are listed with the experimental/intervention group first and the control group second. For multi-arm trials, each value is prefixed with its corresponding group name.

Supplementary Table 4. Funding sources of the included studies

| Ang KK 2014 | We also acknowledge the financial support from the Science and Engineering Research Council of the Agency for Science, Technology and Research, Singapore (Grant number: 092 148 0066) |
| --- | --- |
| Ang KK 2015 | The author(s) disclosed receipt of the following financial support for the research, authorship, and/or publication of this article: The Enterprise Challenge grant, Prime Minister’s Office, Singapore, and in part by the Science and Engineering Research Council of the Agency for Science, Technology and Research, Singapore. |
| Biasiucci A 2018 | This work was partially funded by the EU-ICT FP7-224631 project TOBI (A.A.K. and J.d.R.M.), Swiss NCCR Robotics (J.d.R.M.), the Swiss canton of Valais (R.L., T.S., M.B. and D.V.), and the Wyss Center for Bio and Neuroengineering in Geneva (S.P. and J.d.R.M.). |
| Brunner I 2024 | This study was funded by Helsefonden, Denmark. Grant number 19-B-0147. |
| Cantillo-Negrete J 2025 | The author(s) declare that financial support was received for the research and/or publication of this article. This work was supported by Consejo Nacional de Humanidades, Ciencias y Tecnologías (CONAHCYT) [grant number SALUD-2018-02-B-S-45803]. |
| Cheng N 2020 | This work was funded by the Biomedical Research Council (reference number: 1619077006), Agency for Science, Technology and Research, Singapore. |
| Chung E 2020 | not reported |
| Curado MR 2015 | This work was supported by the German Federal Ministry of Education and Research (BMBF, Förderzeichen 01GQ0831, http://www.bmbf.de/, and DZD, 01GI0925); Deutsche Forschungsgemeinschaft (DFG, http://www.dfg.de/); European Research Council (ERC 227632,http://erc.europa.eu/) and European Union Information and Communication Technologies Framework Programme 7 (ICT-FP7, http://cordis.europa.eu/fp7/ict/) (HUMOUR 231724); TECNALIA (http://www.tecnalia.com/en/); Baden Wuerttemberg Stiftung (ROB-1,http://www.bwstiftung. de/); Natural Science Foundation of China (NSFC31450110072) and Volkswagen Stiftung. MRC was funded by CAPES (Coordination for the Improvement of Higher Level -or Education-Personnel, Brazil, http://www.capes.gov.br/); FLB by CNPq (Brazilian National Counsel of Technological and Scientific Development, http://www.cnpq.br/); MRC, ECG, FLB, WC and OY were funded by DAAD (Deutscher Akademischer Austauschdienst, https://www.daad.org/) scholarship; and GLi was funded by the Interuniversity Center for the Research in Cognitive Processing of Natural and Artificial Systems (ECoNa, http://w3.uniroma1.it/econa/), Sapienza University of Rome. The funders had no role in study design, data collection and analysis, decision to publish, or preparation of the manuscript. |
| Frolov AA 2017 | This study was supported by Russian Ministry of Education and Science, grant RFMEFI60715X0128. |
| Fu J 2023 | This study was supported by the National Natural Integration Project (91948302), the National Key Research and Development Program Project of China (2018YFC2002300), the National Natural Innovation Research Group Project (82021002), the National Natural Science Foundation of China (82202798 and 52005321), and the Shanghai Sailing Program (22YF1404200) |
| Guo N 2022 | date of current version 1 July 2022. This work was supported in part by the Shenzhen-Hong Kong-Macau Technology Research Programme under Grant TypeCSGDX2019081623201196, in part by the Shenzhen Local Science and Technology Development Fund guided by Central Government of China under Grant 2021Szvup130, and in part by the Science and Technology Development Fund, Macau SAR, under Grant 055/2015/A2, Grant 0045/2019/AFJ, and Grant 0018/2019/AKP. (Corresponding author: YongHu.) |
| He J 2025 | This work was supported by Key R & D Program of Shanxi Province No. 2022SF-379 and Xi ‘an Jiaotong University Medical Engineering Interdisciplinary Program No. QYJC05. |
| Ji X 2025 | This study was funded by the National Key Research and Development Program of China (No. 2022YFC2009700), the Key Project of Jiangsu Province’s Key Research and Development Program (No. BE2023023-2), the Competitive Project of Jiangsu Province’s Key Research and Development Program (No. BE2023034), Jiangsu Province Hospital clinical diagnosis and treatment of technological innovation “Open bidding for selecting the best candidates” project (No. JBGS202414), the Open Research Fund of State Key Laboratory of Digital Medical Engineering under (No. 2025-K10), and the Youth Project of the Wuxi Municipal Health Commission (No. Q202414). |
| Kim T 2016 | Not reported |
| Li M 2014 | The authors report no conflicts of interest. This work was granted by the 12th Five-year Plan supporting project of Ministry of Science and Technology of the People's Republic of China (no.: 2013BAI10B03), the Key Projects of Shanghai Science and Technology on Biomedicine (no.10DZ1950800; 13411951001, the Projects of Shanghai Science and Technology on Biomedicine (no.13441902802), the National Natural Science Foundation of China (No.90920014 and 91120305) and the NSFC-JSPS International Cooperation Program |
| Li X 2022 | This work was supported by the Key R&D Program of Guangdong Province, China under grant 2018B030339001 and Key Realm R&D Program of Guangzhou, China under grant 202007030007. |
| Liu X 2023 | This study was supported by the Hebei Province Medical Applicable Technology Tracking Project (GZ2020010). |
| Lu R 2025 | This work was supported by the National Science Foundation of China (82271422, 82372570, T2394534), Shanghai Natural Science Foundation (22ZR1479000), Shanghai Municipal Health Commission (20234Y0043), and Medical Innovation Research Project funded by Shanghai Science and Technology Commission (23Y11900900). |
| Ma ZZ 2024 | This work was supported by the National Key R&D Program of China (Grant Nos.: 2018YFC2001600, and 2018YFC2001604); National Natural Science Foundation of China (Grant Nos.: 81802249, 81871836, 81902301, 82172554); Shanghai Science and Technology Committee (Grant Nos.: 22010504200); Shanghai Rising-Star Program (Grant No.: 19QA1409000); Shanghai Municipal Commission of Health and Family Planning (Grant No.: 2018YQ02, and 201840224). |
| Mihara M 2013 | This work was supported by a Grant-in-Aid for Young Scientists (B) (KAKENHI: 23700500) from JSPS, Japan, a Grants-in-Aid for the Research Committee on the Improvement of Medical Care and the Organization of Supporting Systems for Patients with Rare Diseases, and Grants-in Aid from the Research Committee for Ataxic Disease from MHLW, Japan. |
| Ming Z 2025 | The study was supported by the Xuzhou Key Medical Talents Project (XWRCHT20220045) and National Natural Science Foundation of China |
| Pichiorri F 2015 | This work was supported by the Italian Ministry of Healthcare (RF-2010–2319611) and European ICT Programme Project FP7–224631. This article reflects only the authors’ views, and funding agencies are not liable for any use that may be made of the information contained herein. |
| Ramos-Murguialday A 2013 | This work was supported by the German Federal Ministry of Education and Research (BMBF, Förderzeichen 01GQ0831) as well as the Deutsche Forschungsgemeinschaft (DFG), ), the European Research Council (ERC 227632) and European Union Information and Communication Technologies Framework Programme 7 (ICT-FP7) (HUMOUR 231724), the Intramural Research Program (IRP) of the National Institute of Neurological Disorders and Stroke (NINDS), Bethesda, Maryland, USA, the Center for Neuroscience and Regenerative Medicine (CNRM), Uniformed Services University of Health Sciences, Bethesda, Maryland, US,the Werner Reichardt Centre for Integrative Neuroscience (CIN), Univ. Tuebingen and TECNALIA. Several authors were supported by the DAAD (Deutscher Akademischer Austauschdienst), CNPq (Brazilian National Counsel of Technological and Scientific Development) and CAPES (Coordination for the Improvement of Higher Level -or Education-Personnel, Brazil) and Humbolt Award to LC. The funders had no role in study design, data collection and analysis, decision to publish, or preparation of the manuscript. |
| Wan C 2025 | The authors disclosed receipt of the following financial support for the research, authorship, and/or publication of this article: this study was supported by Jiangsu Provincial People's Hospital, Clinical Diagnosis and Treatment Technology Innovation 'Open bidding for selecting the best candidates' Project (No. JBGS202414), Major sports research projects of Jiangsu Sports Bureau (No. ST242102), Ministry of Industry and Information Technology and National Health Commission High-end Equipment Promotion and Application Project (2024TGYY51), Project supported by the National Natural Science Foundation of China (No.82572957). |
| Wang A 2024 | This work was supported by the National Key R&D Program of China (2018YFC1312903), the National Key Research and Development Program of China (2022YFC3600600), the Training Fund for Open Projects at Clinical Institutes and Departments of Capital Medical University (CCMU2022ZKYXZ009), the Beijing Natural Science Foundation Haidian original innovation joint fund (L222123), the Fund for Young Talents of Beijing Medical Management Center (QML20230505), and the high-level public health talents (xuekegugan-02-47). |
| Wu Q 2020 | This study was supported by Beijing Municipal Natural Science Foundation: Key Technology Research on Ankle Rehabilitation Robot for Motor Nerve Pathways Reconstruction based on Multimodality Information Feedback (L182028), and Beijing Municipal Science and Technology Commission: Research on Rehabilitation Robot for Winter Olympic Ice and Snow Sports Injury (Z181100003118004). No additional external funding was received for this study. The funders had no role in study design, data collection and analysis, decision to publish, or preparation of the manuscript. |

Supplementary Table 5. GRADE assessment for outcomes of BCI-based rehabilitation in post-stroke patients.

| Outcomes | No. of studies | Study design | Risk of bias | Inconsistency | Indirectness | Imprecision | Publication bias | No. of patients | Effect estimate (95% CI) | Certainty |
| --- | --- | --- | --- | --- | --- | --- | --- | --- | --- | --- |
| FMA-UE | 24 | RCT | No | Serious | No | No | No | 1042 | MD = 1.11 (0.63, 1.60) | Moderate |
| ARAT | 8 | RCT | No | No | No | No | No | 524 | MD = 0.21 (0.04, 0.39) | High |
| WMFT | 5 | RCT | Serious | Serious | No | No | Undetected | 425 | MD = 0.94 (0.32, 1.55) | Low |
| MBI | 4 | RCT | Serious | Serious | No | No | Undetected | 153 | MD = 1.17 (0.29, 2.06) | Low |
| FMA-LE | 2 | RCT | Serious | Serious | No | Serious | Undetected | 78 | MD = -0.12 (-0.79, 0.55) | Extremely low |
| BBS | 2 | RCT | Serious | No | No | Serious | Undetected | 35 | MD = 0.54 (0.02, 1.07) | Low |
| TUGT | 2 | RCT | Serious | Serious | No | No | Undetected | 35 | MD = -0.40 (-1.15, 0.34) | Low |

RCT, randomized controlled trial; MD, mean difference; CI, confidence interval; FMA-UE, Fugl-Meyer Assessment-Upper Extremity; FMA-LE, Fugl-Meyer Assessment-Lower Extremity; ARAT, Action Research Arm Test; WMFT, Wolf Motor Function Test; MBI, Modified Barthel Index; BBS, Berg Balance Scale; TUGT, Timed Up and Go Test.

The certainty of evidence was assessed using the GRADE framework. Evidence from randomized controlled trials was initially rated as high certainty and downgraded according to risk of bias, inconsistency, indirectness, imprecision, and publication bias. “Serious” indicates downgrading by one level. Publication bias was considered undetected when the number of studies was small or formal assessment was not applicable.

Supplementary Figure 1. Sensitivity analysis excluding studies at overall high risk of bias for FMA-UE.

Supplementary Figure 2. Sensitivity analysis using a fixed-effect model for FMA-UE.

Supplementary Figure 3. Sensitivity analysis using the Hartung-Knapp-Sidik-Jonkman random-effects method for FMA-UE.

Supplementary Figure 4. Forest plot describes the results of leave-one-out analysis of Fugl-Meyer Assessment-Upper Extremity.

Supplementary Figure 5. Funnel plot describes the publication bias of Fugl-Meyer Assessment-Upper Extremity.

Supplementary Figure 6. Funnel plots for the FMA-UE outcome stratified by external feedback type. (A) Motor-assisted feedback; (B) visual feedback; and (C) multimodal integrated feedback.

Supplementary Figure 7. Forest plot describes the results of Fugl-Meyer Assessment-Upper Extremity in different follow-up.

Supplementary Figure 8. Forest plot describes the results of Fugl-Meyer Assessment-Upper Extremity in different time for per session.

Supplementary Figure 9. Forest plot describes the results of Fugl-Meyer Assessment-Upper Extremity in different external feedback.

Supplementary Figure 10. Forest plot describes the results of Fugl-Meyer Assessment-Upper Extremity among different stroke phases patients.

Supplementary Figure 11. Forest plot describes the results of Fugl-Meyer Assessment-Upper Extremity among different stroke type patients.

Supplementary Figure 12. Forest plot describes the results of Action Research Arm Test.

Supplementary Figure 13. Forest plot describes the results of Wolf Motor Function Test.

Supplementary Figure 14. Forest plot describes the results of Modified Barthel Index.

Supplementary Figure 15. Forest plot describes the results of Berg Balance Scale.

Supplementary Figure 16. Forest plot describes the results of Fugl-Meyer Assessment-Lower Extremity.

Supplementary Figure17. Forest plot describes the results of Timed Up and Go Test.

1.	Chen L, Gu B, Wang Z, Zhang L, Xu M, Liu S, et al. EEG-controlled functional electrical stimulation rehabilitation for chronic stroke: system design and clinical application. Frontiers of Medicine. 2021;15(5):740-9.

2.	Mane R, Chew E, Phua KS, Ang KK, Robinson N, Vinod AP, et al. Prognostic and Monitory EEG-Biomarkers for BCI Upper-Limb Stroke Rehabilitation. IEEE Transactions on Neural Systems and Rehabilitation Engineering. 2019;27(8):1654-64.

3.	Rodríguez-García ME, Carino-Escobar RI, Carrillo-Mora P, Hernandez-Arenas C, Ramirez-Nava AG, Pacheco-Gallegos MdR, et al. Neuroplasticity changes in cortical activity, grey matter, and white matter of stroke patients after upper extremity motor rehabilitation via a brain–computer interface therapy program. Journal of Neural Engineering. 2025;22(2):026025.

4.	Jang Y, Kim T, Lee B. Effects of Brain–Computer Interface-controlled Functional Electrical Stimulation Training on Shoulder Subluxation for Patients with Stroke: A Randomized Controlled Trial. Occupational Therapy International. 2016;23(2):175-85.

5.	Ang KK, Guan C, Phua KS, Wang C, Zhao L, Teo WP, et al. Facilitating Effects of Transcranial Direct Current Stimulation on Motor Imagery Brain-Computer Interface With Robotic Feedback for Stroke Rehabilitation. Archives of Physical Medicine and Rehabilitation. 2015;96(3, Supplement):S79-S87.

6.	Ma Z-Z, Wu J-J, Hua X-Y, Zheng M-X, Xing X-X, Ma J, et al. Evidence of neuroplasticity with brain–computer interface in a randomized trial for post-stroke rehabilitation: a graph-theoretic study of subnetwork analysis. Frontiers in Neurology. 2023;Volume 14 - 2023.

7.	Kumari R, Dybus A, Purcell M, Vučković A. Motor priming to enhance the effect of physical therapy in people with spinal cord injury. J Spinal Cord Med. 2025;48(2):312-26.

8.	Miao Y, Chen S, Zhang X, Jin J, Xu R, Daly I, et al. BCI-Based Rehabilitation on the Stroke in Sequela Stage. Neural Plast [Internet]. 2020 2020; 2020:[8882764 p.].

9.	Fateeva VV, Kushnir AB, Grechko AV, Mayorova LA. [Rehabilitation of patients with post-stroke cognitive impairment using the P300-based brain-computer interface: results of a randomized controlled trial]. Zh Nevrol Psikhiatr Im S S Korsakova. 2023;123(12. Vyp. 2):68-74.

10.	Müller-Putz GR, Daly I, Kaiser V. Motor imagery-induced EEG patterns in individuals with spinal cord injury and their impact on brain-computer interface accuracy. Journal of neural engineering. 2014;11(3):035011.
